# ENABLE 2017, the First EUROPEAN PhD and Post-Doc Symposium. Session 3: In Vitro to In Vivo: Modeling Life in 3D

**DOI:** 10.3390/jpm8020020

**Published:** 2018-05-22

**Authors:** Gianmarco Di Mauro, Ambra Dondi, Giovanni Giangreco, Alexander Hogrebe, Elja Louer, Elisa Magistrati, Meeli Mullari, Gemma Turon, Wouter Verdurmen, Helena Xicoy Cortada, Sanja Zivanovic

**Affiliations:** 1 Institute for Research in Biomedicine (IRB Barcelona), the Barcelona Institute of Science and Technology, Baldiri Reixac 10, 08028 Barcelona, Spain; gemma.turon@irbbarcelona.org (G.T); sanja.zivanovic@irbbarcelona.org (S.Z.); 2 European School of Molecular Medicine (SEMM), via Adamello 16, 20139 Milano, Italy; Ambra.Dondi@ieo.it (A.D.); giovanni.giangreco@ifom.eu (G.G.); elisa.magistrati@ifom.eu (E.M.); 3 Novo Nordisk Foundation Center for Protein Research, University of Copenhagen, Blegdamsvej 3B, DK-2200 Copenhagen N, Denmark; alexander.hogrebe@cpr.ku.dk (A.H.); meeli.mullari@cpr.ku.dk (M.M.); 4 Radboud Institute for Molecular Life Sciences (RIMLS), Radboud University Medical Center, Geert Grooteplein 28, 6525 GA Nijmegen, The Netherlands; Elja.Louer@radboudumc.nl (E.L.); Wouter.Verdurmen@radboudumc.nl (W.V.); Helena.Xicoy@radboudumc.nl (H.X.C.)

**Keywords:** biomedicine, symposium, cancer, stem cells, diabetes, leukemia

## Abstract

The EUROPEAN ACADEMY FOR BIOMEDICAL SCIENCE (ENABLE) is an initiative funded by the European Union Horizon 2020 program involving four renowned European research institutes (Institute for Research in Biomedicine—IRB Barcelona, Spain; Radboud Institute for Molecular Life Sciences—RIMLS, the Netherlands; Novo Nordisk Foundation Center for Protein Research—NNF CPR, Denmark; European School of Molecular Medicine—SEMM, Italy) and an innovative science communication agency (Scienseed). With the aim to promote biomedical science of excellence in Europe, ENABLE organizes an annual three-day international event. This gathering includes a top-level scientific symposium bringing together leading scientists, PhD students, and post-doctoral fellows; career development activities supporting the progression of young researchers and fostering discussion about opportunities beyond the bench; outreach activities stimulating the interaction between science and society. The first European PhD and Postdoc Symposium, entitled “Breaking Down Complexity: Innovative models and techniques in biomedicine”, was hosted by the vibrant city of Barcelona. The scientific program of the conference was focused on the most recent advances and applications of modern techniques and models in biomedical research and covered a wide range of topics, from synthetic biology to translational medicine. Overall, the event was a great success, with more than 200 attendees from all over Europe actively participating in the symposium by presenting their research and exchanging ideas with their peers and world-renowned scientists.

## 1. Introduction

Funded by Horizon 2020, the ENABLE (European Academy for Biomedical Science) project celebrates European research and brings together PhDs and postdocs from all over Europe via activities organized by volunteers and coordinators from the four host institutes—Institute for Research in Biomedicine (IRB) in Barcelona; Radboud Institute for Molecular Life Sciences (RIMLS) in Nijmegen; Center for Protein Research (CPR) in Copenhagen; European School of Molecular Medicine (SEMM) in Milan. In November 2017, the first ENABLE conference took place in Barcelona, Spain. The organization of this conference started almost two years earlier and involved a group of 35 volunteer PhD students and postdocs from the four aforementioned research institutes with the support of institute coordinators and the innovative science communication agency, Scienseed. Like many young scientists nowadays, we felt isolated in our own research areas and wanted to build networks beyond our own fields. This is why we launched ENABLE and what the first ENABLE conference also achieved: it involved young scientists opening the academic world from within and promoted crosstalk between disciplines, collaboration with industry and communication with society at large. The conference in Barcelona was a huge success, with the participation of 272 young researchers from more than 25 countries within the EU and beyond. Companies were also thrilled by our approach, as reflected by our 10 sponsors, which provided more than 60 travel grants, and the more than 20 organizations that were present at our Career Day.

**Figure 1:**
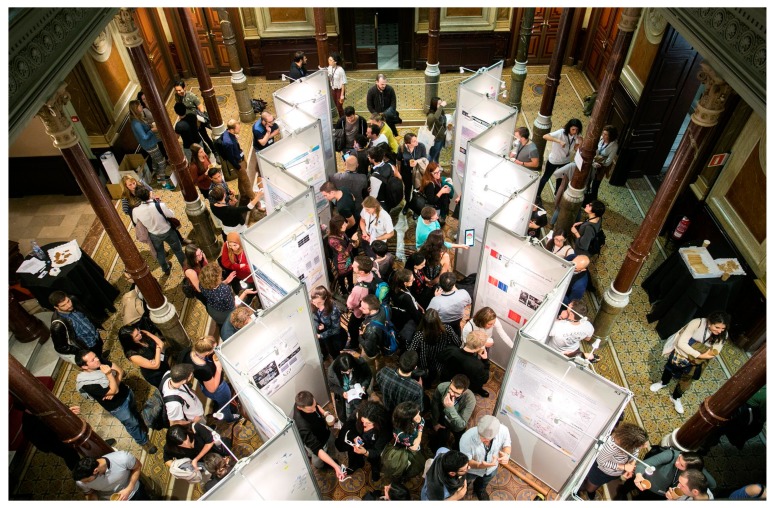
Ongoing vibrant discussions of our participants during the poster session.

The scientific part of the ENABLE conference was a symposium entitled “Breaking Down Complexity: Innovative Models and Techniques in Biomedicine”. It comprised four sessions, spanning from molecular research to clinical research and potential new therapies. In each session, two distinguished keynote speakers presented their research and gave an overview of their work and the directions their fields are taking. Each keynote lecture was followed by two outstanding presentations by postdocs or PhDs selected from among the 272 participants. In addition, 100 posters from a range of biomedical fields were presented by the participants, thus facilitating additional discussion between young researchers with diverse backgrounds (Figure 1). Last but not least, the conference also drew society into the discussion by organizing public debates with experts in the field, inviting school children to take part in scientific problem-solving activities at IRB Barcelona, and giving 24 participating young scientists the opportunity to present their work to the public.

## 2. Session 3: In Vitro to In Vivo: Modeling Life in 3D—Summary

Entitled ‘In Vitro to in Vivo: Modeling Life in 3D’, session three began with a keynote lecture by Dr. **Kristina Havas Cavalletti** (young group leader, the FIRC Institute of Molecular Oncology (IFOM), Milan, Italy), who talked about her team’s work on metabolism and therapy resistance in breast cancer models. She discussed the need to identify and study therapy-resistant tumor cells in minimal residual disease states. She explained the mouse models and organoid cultures that her lab uses for studying therapy-resistant breast cancer cells using a variety of systems biology approaches. Her team discovered a transcriptional state of residual cells that differed from healthy cells and primary tumors. The transcriptional changes suggested an altered lipid metabolism, which was confirmed by further biochemical experiments. The residual cells showed enhanced oxidative DNA damage, which was reduced by inhibiting fatty acid synthesis or transport into mitochondria. She also showed that reactive oxygen species (ROS) inhibition reduced the recurrence of tumors. She concluded that both lipid metabolism and ROS are promising therapeutic targets for preventing the survival of therapy-resistant tumor cells.

The next speaker was Dr. **Juliette Mouriès** (Post-Doctoral researcher, European Institute of Oncology, Milan, Italy), who gave a short talk on the relationship between gut vascular barrier disruption and the onset of type 2 diabetes. She showed that a high-fat diet induced barrier disruption before diabetes onset in wild-type mice. In contrast, a mouse model where the gut vascular barrier cannot be disrupted showed resistance to type 2 diabetes, which indicates a direct link between the two. The second short talk was given by **Fernando Sotillo** (PhD student, IRB Barcelona, Spain), who demonstrated that metabolic dysfunction can drive immune and hematologic complications in a mouse model of Lysinuric Protein Intolerance, a rare autosomal disease caused by mutations in the *SLC7A7* gene. He studied the role of this gene in a tamoxifen-inducible KO mouse model and found that aberrant iron accumulation may explain some of the immune complications observed.

In his keynote lecture, Dr. **Kim Jensen** (Associate Professor, University of Copenhagen, Denmark) discussed his team’s work on the molecular mechanisms that govern cell fate specification, tissue maturation, and cellular plasticity in the developing and adult intestinal epithelium. He explained that adult stem cells located at the bottom of the crypts of Lieberkühn are responsible for the life-long replenishment of the epithelium lining of the intestinal wall. These stem cells reside in specialized niches surrounded by secretory Paneth cells, basement membrane proteins, and fibroblasts. In his lecture, he combined insights from a large variety of techniques, including organotypic cultures, flow cytometry, fluorescence-activated cell sorting, RNA sequencing, and immunohistochemical staining of mouse and human tissues. Taken together, the findings of Jensen’s lab support the notion that stem cell identity is an acquired rather than a hard-wired trait and that cell fate in this regard is dynamically regulated to cater for the immediate needs of the tissue. He explained the important role of yes-associated protein/transcriptional coactivator with PDZ-binding motif (YAP/TAZ) activation in the process and also highlighted phenotypical differences between fetal and adult epithelium that emphasize the dynamic nature of cell fate. His take-home message was that cellular context, such as matrix and environment, must always be considered when studying (stem) cell fate.

The next speaker was **Paolo Falvo** (PhD student, European Institute of Oncology, Milan, Italy), who gave a short talk on the role of obesity in the development of acute promyelocytic leukemia. Specifically, he showed that a high-fat diet in transgenic mice constitutively expressing promyelocytic leukemia/retinoic acid receptor alpha (PML/RARα) in the hematopoietic system, a model for acute promyelocytic leukaemia, resulted in more DNA damage in hematopoietic stem cells and in enhanced clonogenic activity, thus facilitating tumor development. Subsequently, **Jordi Badia-Ramentol** (PhD student, IRB Barcelona, Spain) showed that targeting transforming gowth factor-β (TGF-β) in the tumor microenvironment is a promising approach in metastatic colorectal cancer. He showed the immunosuppressive role of TGF-β using a novel mouse model, which reproduces liver metastases in an immunocompetent mouse strain, and its potential to study the interplay between TGF-β and each component of the tumor microenvironment.

## 3. Conclusions and Future Perspectives of ENABLE

The first ENABLE conference was a success: 35 PhD and postdoc volunteers from four European research institutes, with support from the institute coordinators and Scienseed, organized an event in which 272 young researchers from over 25 countries presented and shared their science and experiences in scientific talks, poster sessions, master classes, general public talks and evening activities. More than 60 attendees were given the opportunity to participate through the award of a travel grant funded by one of our 10 sponsors. The symposium, entitled “Breaking down complexity: innovative models and techniques in biomedicine”, was created to cover a broad range of topics in biomedical research, to encourage participation and the exchange of ideas, and to promote future collaborations among young scientists. 

In order to include multiple research areas, there was a scientific program with four sessions. The first, entitled “Building the foundations of biology: synthetic and cellular research”, had Prof. Martin Hanczyc (University of Trento, Italy) and Prof. Elaine Fuchs (The Rockefeller University, NY, USA) as keynote speakers and included short talks on death-associated protein kinase 1 (DAPK) regulation, nanoscale redistribution of N-methyl D-aspartate (NMDA) receptors in autoimmune encephalitis, targets associated with metabolic reprogramming in hematological malignancies, and the mechanisms regulating aortic arch development. The second session, “The OMICS revolution: understanding the layers of life”, had Prof. Johan Auwerx (Ecole Polytechnique, Lausanne, Switzerland) and Prof. Ruedi Aebersold (ETH Zurich, Switzerland) as keynote speakers and included short talks on proteomics to study the role of polycomb repressive complex 2 (PRC2) in embryonic stem cells, single-cell sequencing to reconstruct the cell lineages of whole adult animals, the effects of endocrine-disrupting chemicals on development, and on quantitative proteomics to study fibrotic networks. The third session, “In vitro to in vivo: modeling life in 3D”, had Prof. Kristina Havas Cavalletti (IFOM, Milan, Italy) and Dr. Kim Jensen (Biotech Research & Innovation Centre (BRIC), Copenhagen, Denmark) as keynote speakers and included short talks on gut vascular barrier disruption and type 2 diabetes, the link between metabolic dysfunction and immune complications in lysinuric protein intolerance, the role of obesity in the development of acute promyelocytic leukemia and the role of TGF-β pathway in the microenvironment of colorectal cancer metastasis. The fourth and final session, “From discovery to cure: the future of therapeutics”, had Prof. Eytan Ruppin (University of Maryland, Center for Bioinformatics and Computational Biology (CBCB), USA) and Prof. Christian Brander (IrsiCaixa, Barcelona, Spain) as keynote speakers and included short talks on the role of miR27a as a tumor suppressor, photodynamic cancer therapy, real-time in vivo monitoring of transplanted islets, and a high-density lipoprotein nanodisc for the potential treatment of cerebral β-amyloidosis.

The success of the event was confirmed by the satisfaction scores (out of 5) given by the participants. In this regard, they gave the 2017 ENABLE symposium 4.4, the general topic of the symposium 4.1, and the keynote talks 4.4. The favorite part of the symposium was “Tapas with the speakers” (score of 4.5), an activity that allowed the participants to interact with the keynote speakers in an informal setting while enjoying some typical Spanish food. The satisfaction of the attendees was also reflected by comments made on the evaluation form, such as “The option of travel grants is amazing and the general idea of the symposium is great. Great speakers, amazing food, nice event. Congratulations”, “I think that the ENABLE project is an amazing idea. It is a little bit different than other conferences because of the career day. The fact that it was organized by PhD students is really interesting!” and “On the all, the symposia was super nice and it was extremely great to really discuss science on a reality level. No one wanted to show off or pretended to be the best scientist in the world and this was awesome.”

To conclude, the symposium brought together renowned scientists with young scientists and can be considered a huge success. The enthusiasm of the participants and the positive feedback received after the event underscore this notion and indicate that the ENABLE conference series has got off to an excellent start, with all eyes now focused on the 2018 event in Copenhagen.

The second symposium of the ENABLE series will be hosted by the Novo Nordisk Foundation Center for Protein Research (CPR, University of Copenhagen, Denmark), one of the partner institutions of the ENABLE consortium. It will take place November 6–9, 2018 at the Maersk Tower in Copenhagen. 

Entitled “The promise of future medicine: from research to therapy”, the symposium will explore state-of-the-art biomedical research from basic science to clinical practice and patient outcome. By bringing together 300 PhD students and postdocs, as well as nine eminent keynote speakers from diverse research fields, the next ENABLE symposium seeks to foster a multidisciplinary environment and crosstalk between biomedical disciplines. The following speakers have already confirmed their participation: Helen Lee (Cambridge University, Cambridge, UK); Giuseppe Testa (European Institute of Oncology, Milan, Italy); Nazneen Rahman (Institute of Cancer Research, London, UK); Klaus Pantel (Institute of Tumour Biology, University Medical Centre Hamburg-Eppendorf, Hamburg, Germany); Michel Morange (Institute for History and Philosophy Science And Technology (IHPST), Paris, France); Andrea Bertotti (Institute for Research and Health Care (IRCCS) Candiolo, Italy); Matthew Wood (University of Oxford, Oxford, UK). 

Apart from the scientific symposium, a Career Day is foreseen to allow participants to broaden their career perspectives. This activity will involve chats with professionals, high-quality workshops, and an Opportunity Fair, which will allow participants to come into direct contact with companies belonging to a variety of sectors. To support the participation of young researchers from all over Europe, our sponsors will provide about 40 travel grants to cover the registration fee and travel and accommodation expenses. Updated information on the event can be found on our website (https://enablenetwork.eu/). 

We look forward to the 2018 symposium and are confident that ENABLE will foster the establishment of a network that promotes efficient and synergistic scientific exchange among researchers throughout Europe.

